# The Muscle Function Deficit Concept and Inflammaging

**DOI:** 10.3390/biomedicines14020383

**Published:** 2026-02-06

**Authors:** Giada Mariano, Matteo Candeloro, Raffaello Pellegrino, Roberto Paganelli, Angelo Di Iorio

**Affiliations:** 1 Department of Innovative Technologies in Medicine & Dentistry, University “G. d’Annunzio”, 66100 Chieti, Italy; giada.mariano@studenti.unich.it (G.M.); matteo.candeloro@unich.it (M.C.);; 2 Department of Medicine and Surgery, Università LUM “Giuseppe Degennaro”, Casamassima, 70010 Bari, Italy; 3 Internal Medicine, Saint Camillus International University of Health and Medical Sciences, 65100 Rome, Italy; 4 SYNERGO Institute of Clinical Immunotherapy and Advanced Biological Treatments, 66100 Pescara, Italy

**Keywords:** skeletal muscle function deficit, inflammaging, sarcopenia

## Abstract

Aging-related muscle dysfunction has been conceptualized through the model of sarcopenia, but it embraces several other characteristics, e.g., dynapenia, myosteatosis, and powerpenia. Our perspective reframes muscle aging from a different point of view, the Skeletal Muscle Function Deficit (SMFD), a unifying approach that integrates muscle quality and mass into a single functional definition. An SMFD score has been adopted in the InCHIANTI study against many geriatric outcomes, such as risk of disability, physical performance, hospitalizations and falls, and incidence of major diseases, highlighting its potential value as a primary indicator of muscle failure and/or of healthy aging. At the core of SMFD lies inflammaging, the chronic, low-grade, age-related inflammation, linking functional outcomes to muscular and neural aging. Inflammatory mediators alter the anabolic/catabolic balance, accelerate myosteatosis, impair neuromuscular junction, and influence denervation. These findings support the idea of a common pathway that links neuro-muscular deficit and inflammation, which simultaneously targets cortical motor circuits, spinal motor neurons, peripheral nerves, and muscle fibers. The SMFD approach facilitates early detection, risk stratification, and possible intervention for muscle deterioration with aging.

## 1. Introduction

The aging process induces a progressive decline of skeletal muscle, involving not only reductions in muscle mass but also impairments in strength [1], power [2], muscle quality [3], fatigability, and neuromuscular control [4] features that are not fully captured by the traditional definition of sarcopenia [1,5,6]. This multidimensional deterioration contributes to functional limitations and vulnerability to disabling conditions. Importantly, declines in muscle function, rather than loss of muscle mass per se, are the primary determinants of reduced quality of life, loss of independence, and impaired self-sufficiency in older adults [7,8].

Furthermore, sarcopenia is parallel to the physiological cognitive deterioration during senescence progression [9]. General deterioration starts in the fifth decade of life, with muscle loss of approximately 0.8–2% per year [2]. There is an estimated 30% reduction in muscle mass and a 20% reduction in muscle area between subjects in their 20s versus 80-year-old adults, and about 20% of elderly subjects are in a frail state [10,11]. However, due to its focus on muscle mass and strength loss, the sarcopenia approach has several limitations in predicting functional decline [12,13].

The early definition of sarcopenia centered only on the reduction of muscle mass [14], ignoring the multidimensional nature of muscle aging, and the later inclusion of muscle strength as a diagnostic criterion in the model recognized the functional consequences of the muscle impairment [6,15]. A useful way to understand the European Working Group on Sarcopenia in Older People (EWGSOP) decision to include physical performance in the definition of sarcopenia is to draw a parallel with heart failure. In heart failure, left ventricular ejection fraction provides an anatomical and functional measure of pump dysfunction, but clinical severity and prognosis are stratified according to NYHA classes, which capture how much that dysfunction limits daily activities. In an analogous manner, EWGSOP2 places low muscle strength at the cornerstone of sarcopenia and uses physical performance to grade severity, acknowledging that the clinical syndrome is expressed as limitation in mobility and daily function rather than as low lean mass alone [15,16,17]. However, sarcopenia is detected late in the continuum of muscle aging, probably when the effect of muscle dysfunction is practically irreversible [2]. This could help to explain the non-univocal results of clinical trials and observational studies that have assessed the effects of nutritional supplementation or physical activity [18,19,20].

The delayed identification of sarcopenia may partly explain the proliferation of related terms aimed at capturing specific aspects of age-related muscle dysfunction, including sarcopenic obesity (the coexistence of reduced muscle mass and excess adiposity) [21], dynapenia (loss of muscle strength alone) [22], powerpenia (age-related loss of muscle power and explosiveness) [2,23], and myosteatosis (fat infiltration within skeletal muscle, reflecting impaired muscle quality) [24,25]. While each definition highlights a relevant phenotype, their coexistence underscores the fragmented conceptualization of a single, multifaceted biological process.

**Figure 1:**
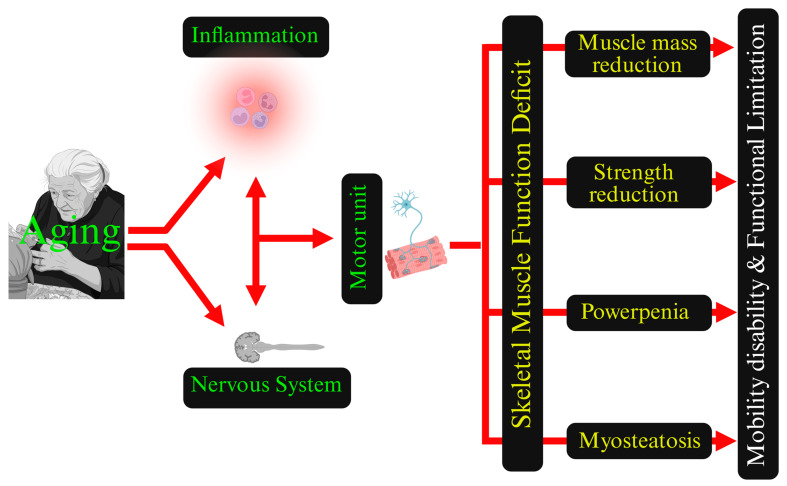
Integrated model of Skeletal Muscle Function Deficit (SMFD). SMFD is presented as an integrated functional entity consisting of four key domains of muscle aging: muscle area (mass), muscle strength, muscle power, and myosteatosis (fat infiltration). Reciprocal inter-relations are shown by bidirectional red arrows, to indicate that each of these domains cumulatively contributes to functional decline. The arrows indicate that aging and chronic low-grade inflammation are the biological drivers that act simultaneously on these domains and accelerate neuromuscular deterioration. The clinical downstream consequence is depicted on the right, where impaired muscle function leads to mobility limitation as clinical endpoint. This scheme is representative of a paradigm shift, moving from isolated phenotypes to an integrated framework where muscle mass, quality, and performance co-determine functional capacity. Figure is created in BioRender. Di Iorio, A. (2025) https://BioRender.com/q8vbwc4, accessed on 26 January 2026.

While conceptually interesting, this fragmentation results in obscuring the interconnected nature of the problem. In this perspective, re-focusing on skeletal muscle function as a whole and on its early, often subtle, alterations in power, coordination, fatigability, and muscle quality may represent a step toward the future instead of a “return to the past” [26]. This approach redefines a syndromic, integrative view of muscle failure. Within this framework, SMFD offers an umbrella concept that embeds all the aging muscle definitions into a single construct centered on function rather than on single structural or performance metrics (Figure 1) [26]. Yet, to the best of our knowledge, SMFD has remained largely a theoretical proposal and has not been fully translated into an operational model with explicit criteria, staging, and thresholds for clinical or research use; briefly, muscle action effectiveness lacks a comprehensive metric.

This viewpoint aims to examine age-related muscle deterioration through an integrated neuromuscular and immune approach, moving beyond the traditional, mass-centered definition of sarcopenia.

## 2. SMFD Operational Definition and Validity

The InCHIANTI study (Invecchiare in Chianti: aging in the Chianti area) is a study of the factors contributing to the decline of mobility in later life; the main purpose is to establish a minimum set of clinical variables that allow detection of the underlying causes of walking difficulties [27]. The study was designed by the Laboratory of Clinical Epidemiology of the Italian National Research Council on Aging, (INRCA, Florence, Italy) in a partnership with the local administrators of Greve in Chianti and Bagno a Ripoli, two small towns in the countryside of the Tuscany area (Supplementary Figure S1). The InCHIANTI study translated the theoretical construct of SMFD into an operational composite score and validated it against major geriatric outcomes [28]. While that work primarily addressed the methodological and prognostic validity of SMFD, the present viewpoint extends these findings by focusing on the biological and pathophysiological framework underlying SMFD, with particular attention to the role of inflammaging and neuromuscular integrity.

SMFD was quantified as a composite 0–20 score integrating lower-limb muscle mass, muscle density, grip strength, and leg power, each transformed into sex-specific quintiles with an additional “unable to perform” category and followed for nine years (3196 observations from 1035 older subjects). As the SMFD score declined over time, there was a parallel and consistent worsening of functional outcomes: higher SMFD scores were independently associated with a substantially lower risk of disability in basic and instrumental activities of daily living, better physical performance (higher Short Physical Performance Battery, SPPB), and a lower likelihood to be classified as Frail, according to Fried’s frailty score [29,30].

SMFD should be distinguished from frailty, although the two constructs partially overlap. Frailty represents a global clinical syndrome of multisystem vulnerability [31], whereas SMFD is a muscle-specific construct focused on neuromuscular function and muscle quality [26]. Unlike Fried’s frailty score [30], SMFD captures early, preclinical alterations in muscle performance and power that may precede the clinical manifestation of frailty. In this sense, SMFD may modulate the development of physical frailty rather than be an alternative frailty definition [28,32].

Also, for “harder” outcomes, SMFD showed a significant inverse association with the risk of hospitalization and with the number of falls during follow-up, indicating that preserved muscle function is linked not only to better performance measures but also to reduced acute clinical instability. Moreover, higher SMFD scores were inversely associated with the prevalence and incidence of major chronic conditions, particularly Parkinson’s disease, stroke, and hip osteoarthritis, suggesting that SMFD may capture early neuromuscular and musculoskeletal vulnerability that precedes overt clinical disease. Although mortality was not a central endpoint in this analysis, the strong and independent associations of SMFD with disability, performance, hospital use, and incident disease support its potential as an early indicator of survival and healthy longevity. Overall, the work shows that the SMFD score, combining quantitative and qualitative muscle markers, is a robust predictor of several adverse outcomes in older adults and represents a pragmatic, function-oriented tool for risk stratification in gerontological research and in geriatric settings [28,32].

## 3. SMFD and Inflammation

Several complex and multifactorial causes have been invoked in the etiology of SMFD [26]. Inflammation is considered one of the major determinants of muscle aging, modulating many other pathophysiological processes [33,34,35].

For instance, myosteatosis represents a prominent aspect of the aging muscle, characterized by the infiltration of adipose tissue into and within the muscle fibers [3]. This impairs muscle quality but also exacerbates the inflammatory status and establishes a feedback loop, which accelerates muscle degradation. Similarly, mitochondrial dysfunction, which can be triggered by chronic inflammation, further impairs muscle regeneration and energy production, adding to muscle weakness and fatigability [36,37]. These interconnected processes underline the requirement for a multidimensional approach toward the prevention and treatment of SMFD.

In parallel to Fried’s definition of physical frailty [29], inflammaging (a chronic, low-grade inflammatory state) [38] can be described as a transition from “immunological homeostasis symphony to immunological cacophony”. Inflammaging could, therefore, represent the unifying mechanism linking declines in the different components of the aging muscle: muscle mass, strength, quality, and power [33].

A hallmark of inflammaging is the chronic elevation of pro-inflammatory cytokines, including IL-6, TNF-α, and IL-1β, largely derived from senescent immune cells, dysfunctional adipose tissue, and other aging tissues [39,40]. IL-6 acts with context-dependent effects: when transiently released by contracting skeletal muscle during physical activity, IL-6 acts as a myokine with metabolic and regenerative properties [41,42,43]; conversely, its persistent low-grade elevation in aging reflects a pro-inflammatory state associated with catabolic signaling, impaired muscle regeneration, and functional decline [44,45]. This inflammatory environment chronically alters the balance between anabolic and catabolic pathways [46]: sustained activation of NF-κB and JAK/STAT3 [47], together with inhibition of PI3K/Akt/mTOR signaling [48], shifts the equilibrium toward protein degradation [49], reduced myofibrillar synthesis [50], impaired satellite-cell activation [51], and anabolic resistance to exercise and amino acid intake [52]. In parallel, inflammation promotes denervation and neuromuscular junction deterioration, accelerating losses in strength and power [33,53]. At the tissue level, inflammation promotes adipogenic differentiation of mesenchymal progenitors and accumulation of intra- and intermuscular fat (myosteatosis), worsening muscle quality [54]. Activation of the NLRP3 inflammasome further increases IL-1β production and inflammatory signaling, creating a self-sustaining loop of damage and cellular senescence continuously feeding the inflammation [55,56].

Anti-inflammatory mediators such as IL-10, primarily produced by regulatory T cells and monocyte/macrophage lineages with a minor contribution from skeletal muscle, and adiponectin can exert partially protective effects in frail older individuals [57]; however, their activity appears insufficient to counterbalance the prevailing pro-inflammatory burden [58]. Overall, inflammaging emerges as a central biological process consistently associated with the progressive loss of muscle structure, function, and neuromuscular resilience captured by SMFD. However, inflammaging should be regarded as a key contributor within a multifactorial network rather than as a single proven causal driver [59].

The biological effect of chronic inflammation extends throughout the motor system, supporting the concept of a neuro-muscular inflammaging axis linking central and peripheral neural system susceptibility with progressive loss of muscle function [60]. Rather than representing entirely separate trajectories, muscle dysfunction, peripheral nerve deterioration, and central nervous system changes appear to share a common inflammatory milieu [33,34]. While biologically plausible, this neuro-muscular inflammaging axis should be interpreted as a hypothesis supported by converging associative and longitudinal evidence, rather than as a fully established causal pathway [61].

In the Mugello Study, a survey with the purpose of identifying biological and clinical markers of quality of life in nonagenarians [62], peripheral immune activation markers (particularly monocyte-related ratios) were associated with dementia diagnoses in the oldest-old, showing that continued inflammatory pressure shapes the vulnerability of the central nervous system (CNS), microglial priming, and neural network integrity [63]. This reflects a shift toward a pro-inflammatory milieu that permeates the blood–brain barrier, alters neuroimmune communication, and promotes neurodegenerative mechanisms in regions essential for motor planning, coordination, and executive control [64]. Moreover, the InCHIANTI study shows that inflammatory markers predict linear declines in peripheral motor nerve conduction velocity, independently of age and comorbid conditions [33]. We hypothesized that inflammation-induced myelin damage, axonal metabolic stress, and impaired Schwann-cell support were pivotal to motor nerve conduction reduction [65,66]. This decline in peripheral nerve function shortly predates or accompanies the impairment in muscle strength, coordination, and power [33,66].

Taken together, these findings support a model in which age-related inflammatory activity exerts wide effects on neurological and motor control. Therefore, muscle decline is not simply a downstream victim of the aging motor neuron, but they are both targets of inflammaging [67].

## 4. Conclusions

The theoretical model of the SMFD, as operationalized in the InCHIANTI study, redefines the muscle-age-related process of decline under a wider umbrella. This approach offers a more consistent and clinically meaningful way to understand and study muscle aging, compared to the more limited definitions of sarcopenia, dynapenia, powerpenia, myosteatosis, and related/similar entities.

Instead of treating these muscle-aging markers as separate entities, SMFD brings them back into a single, function-centered construct. The model better reflects the real-life condition of older adults, in whom physical function, mobility, power, and resilience may decline long before muscle mass crosses any threshold. Within this framework, inflammaging and nervous system failure emerge as biological cornerstones of the different dimensions of SMFD.

This perspective proposes the use of SMFD, switching from a purely conceptual definition to an operational, measurable, and function-oriented nosological entity. Such a framework can be used to quantify early vulnerability, to stratify risk, and to explain why late, mass-based definitions of sarcopenia have often produced inconsistent results in trials of nutritional supplementation and physical activity programs.

Looking forward, research should refine SMFD measurement, identify clinically feasible proxies, and test interventions explicitly designed to modulate inflammation and neuro-muscle function. Ultimately, integrating SMFD into geriatric practice may open a path from late recognition of an irreversible decline to earlier, mechanism-based strategies for maintaining mobility, independence, and healthy longevity.

## 5. Limitations and Future Directions

The evidence supporting the construct of SMFD derives primarily from the theoretical conceptualization by Rosaly-deAraujo [26], and by a single, well-characterized population-based cohort [28], which may limit generalizability across ethnicities and healthcare settings. Moreover, the observational design precludes definitive causal inference regarding the role of inflammation and neuromuscular decline. Future studies should aim to externally validate SMFD in independent cohorts, identify clinically feasible proxies for its assessment, and test interventions specifically targeting inflammatory and neuromuscular pathways.

## Data Availability

No new data were created or analyzed in this study. Data sharing is not applicable to this article.

## Supplementary Materials

The following supporting information can be downloaded at: https://www.mdpi.com/article/10.3390/biomedicines14020383/s1, Figure S1: An outline of the InChianti (Tuscany, Italy) study.
