# Supplementary material for: The Muscle Function Deficit Concept and Inflammaging
Source: Biomedicines. 2026 Feb 6;14(2):383. doi: 10.3390/biomedicines14020383 (PMC12938328; doi:10.3390/biomedicines14020383)
Supplement: Supplementary file 1 [file biomedicines-14-00383-s001.zip › biomedicines-4088762-supplementary.pdf]

Supplementary Figure 1

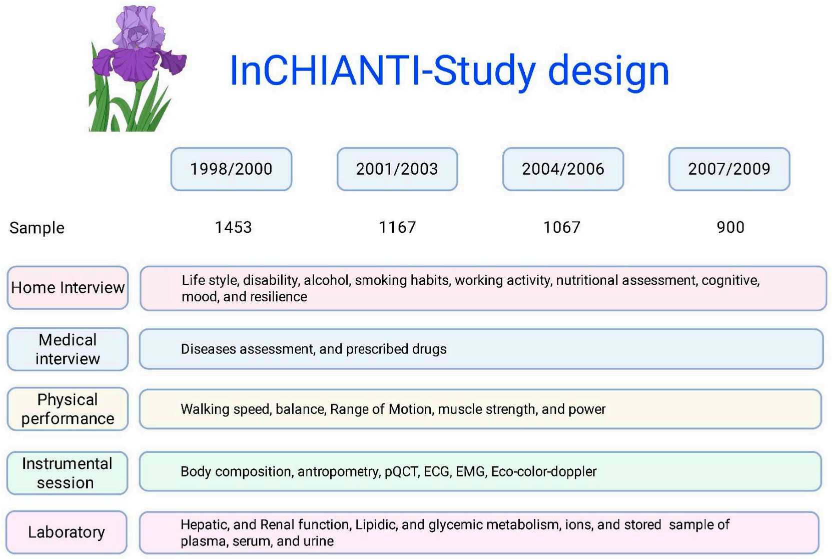

Supplemental Figure S1 : An outline of the InChianti (Tuscany, Italy) study.

The InCHIANTI ("Invecchiare in Chianti": aging in the Chianti area) is a study of the factors contributing to the decline of mobility in late life conducted in two small towns in the countryside of the Tuscany area (Greve in Chianti and Bagno a Ripoli), described in (1). The study started in 1998 and was completed in the first months of 2000. 1453 subjects were randomly selected from the population registry of the two sites. The study consisted of a home interview (family composition and social networks, cognitive and depressive symptoms, ability to perform daily life activities, foot problems, falls and fear of falling, incontinence, quality of sleep, and food intake), an instrumental session (a peripheral quantitative computed tomography, a surface electroneurography, a standard electrocardiogram, an ultrasound color doppler examination of the carotids, vertebral arteries and veins of the lower limbs), a medical interview with a trained geriatricians to explore and assess major medical conditions and drugs prescribed and their consumption. Physical performance objectively assessed physical function, muscle and power strength, joint range of motion and walking abilities. Blood samples were drawn and stored with also an aliquot of 24-hour urine collection. The InCHIANTI follow-up was conducted every three years, including the same items performed at baseline. Figure is created in BioRender. Di iorio, A. (2026) https://BioRender.com/imu286e.

References

1. Ferrucci L, Bandinelli S, Benvenuti E, et al. Subsystems contributing to the decline in ability to walk: Bridging the gap between epidemiology and geriatric practice in the InCHIANTI study. J Am Geriatr Soc. 2000;48(12):1618-1625. doi:10.1111/j.1532-5415.2000.tb03873.x.
